# NEUROBEHAVIOR OF PRETERM, SMALL AND APPROPRIATE FOR GESTATIONAL AGE NEWBORN INFANTS

**DOI:** 10.1590/1984-0462/;2018;36;4;00006

**Published:** 2018

**Authors:** Inalu Barbosa da Silva, Paola Andrade Gomes da Cunha, Maria Beatriz Martins Linhares, Francisco Eulógio Martinez, José Simon Camelo

**Affiliations:** a Universidade de São Paulo - campus Ribeirão Preto, Ribeirão Preto, SP, Brasil.

**Keywords:** Infant, premature, Infant, small for gestational age, Child development, Recém-nascido prematuro, Recém-nascido pequeno para a idade gestacional, Desenvolvimento infantil

## Abstract

**Objective:**

To compare the neurobehavioral development of preterm infants with postconceptional age between 32 and 36 weeks and 6 days, according to the adequacy of the weight for the gestational age at birth.

**Methods:**

A cross-sectional study was performed comparing two independent groups. The 55 preterm infants who were included in the sample were hospitalized in a neonatal intermediate care unit and were evaluated using the Neurobehavioral Assessment of the Preterm Infant (NAPI) at the postconceptional age between 32 and 36 weeks and 6 days and compared according to the adequacy of the weight for the gestational age. In addition to the comparison between the groups, infants who were born small for gestational age (SGA) and those ones adequate for gestational age (AGA) were also compared, considering the type of intrauterine growth. The following instruments were used: NAPI, anamnesis script, Brazilian Economic Classification Criteria, and medical records.

**Results:**

Infants were born with mean gestational age of 32.0 weeks, with the postconceptional age and postnatal age of 34.8 weeks and 19.5 days, respectively. The sample consisted of 55% of female infants. The results did not show any differences in NAPI domains between SGA and AGA groups, neither in the subgroups of SGA babies with symmetric or asymmetric growth.

**Conclusions:**

There was no difference between SGA and AGA babies in relation to neurobehavioral development evaluated before reaching term.

## RESUMO

**Objetivo:**

Comparar o desenvolvimento neurocomportamental de bebês pré-termo com idade pós-concepcional entre 32 e 36 semanas e 6 dias, de acordo com a adequação do peso para a idade gestacional ao nascer.

**Métodos:**

Realizou-se um estudo transversal de comparação entre dois grupos independentes. Os 55 bebês prematuros que compuseram a amostra estavam internados em uma unidade de cuidados intermediários neonatais e foram avaliados por meio de *Neurobehavioral Assessment of the Preterm Infant* (NAPI) com idade pós-concepcional entre 32 e 36 semanas e 6 dias e comparados de acordo com a adequação do peso para a idade gestacional. Além da comparação entre os grupos, bebês nascidos pequenos para a idade gestacional (PIG) e os adequados para a idade gestacional (AIG) também foram comparados, considerando o tipo de crescimento intrauterino. Os seguintes instrumentos foram utilizados: NAPI, roteiro de anamnese, Critério de Classificação Econômica Brasil, da Associação Brasileira de Empresas de Pesquisa (ABEP), e prontuário médico.

**Resultados:**

Na população de estudo, a idade gestacional média foi de 32,0 semanas, enquanto a idade pós-conceptual e cronológica à avaliação foi de 34,8 semanas e 19,5 dias, respectivamente, sendo 55% dos bebês do sexo feminino. Não houve nenhuma diferença nos domínios do NAPI entre os grupos PIG e AIG, nem nos subgrupos de bebês PIG classificados segundo o crescimento em simétrico ou assimétrico.

**Conclusões:**

Não houve diferença entre os bebês PIG e AIG em relação ao desenvolvimento neurocomportamental avaliado antes de chegar ao termo.

## INTRODUCTION

Fetal growth has been the focus of attention for some health professionals and researchers who work with childhood development, since it is an important parameter to assess neuropsychomotor development. It is important to mention that intrauterine growth restriction (IUGR) and the condition of being small for gestational age (SGA) are not equivalent. SGA children are not only those who were born with IUGR, but also children who are small in terms of constitution. Many studies relate the fact of being SGA with increasing mortality and morbidity in comparison to infants who were born adequate for gestational age (AGA).^1^ ^,^ ^2^ ^,^ ^3^

Pinello et al*.* ^4^ studied the visual performance, the psychomotor performance and the cognitive development of preterm children who were SGA and AGA, and showed that the SGA aged one year of corrected age were more prone to presenting with low visual performance and abnormal cognitive development. In the long term, other authors observed neurological development deficit in premature infants born SGA at the age of five years, in comparison to premature infants born AGA, and these deficit rates were mostly associated with microcephaly^5^ However, the studies are controversial when they state that the condition of premature SGA birth increases the risks for problems in neuropsychomotor development in the short, medium or long term.^4^ ^,^ ^6^ ^,^ ^7^ ^,^ ^8^ These studies assessed only preterm newborns (PT-NB) after reaching the age of term or after that, and there are not many investigations comparing the neurodevelopment of premature newborns SGA and AGA before they reach 37 weeks of postconceptional age.

The neurobehavioral assessment in the neonatal period is the first opportunity to understand the contribution of the newborn (NB) for the interactions that will be established with the environment. In this sense, it is possible to mention the *Neurobehavioral Assessment of the Preterm Infant* (NAPI), elaborated to assess the neurobehavioral maturity of PT-NB before reaching term. Some studies that used this instrument demonstrated its predictive validity.^9^ ^,^ ^10^ Constantinou et al*.* ^11^ found correlation between low scores in NAPI at the postconceptional age of 36 weeks, low scores in the *Bayley Infant Neurodevelopmental Screener* (BINS), with 12 months, and also in the *Bayley Scales of Infant and Toddler Development* (BSID II) in the ages of 18 and 30 months, in the group of children born weighting <1,000 g, in comparison to the group of children born between 1,000 and 1,500 g.

NAPI was developed in three stages, which included a pilot study, an exploratory study and a validation study. During its construction, 990 preterm infants were assessed and divided into three groups. To determine its clinical validity, the children were assessed by the *Neonatal Medical Index* (NMI). Then, the data were compared statistically with the scores obtained in NAPI, in order to understand if the instrument distinguishes the behavioral development of infants who have had serious clinical complications from those who did not have these complications or not.^10^ ^,^ ^12^

Since the period before term is considered essential for the understanding of the initial interaction between the NB and the environment, and, besides, there are few investigations comparing the initial neurodevelopment between premature newborns SGA and AGA, this study proposed to investigate the hypothesis that premature infants born SGA present with worse neurobehavioral performance in relation to AGA infants, assessed by the NAPI before reaching 37 weeks of postconceptional age. Therefore, our objective was to compare the neurodevelopment of premature NB with postconceptional age between 32 and 26 weeks and 6 days, distinguished in groups according to the adaptation of weight for gestational age.

## METHOD

This is a cross-sectional study approved by the Research Ethics Committee of Hospital das Clínicas, in the Medical School of Ribeirão Preto, Universidade de São Paulo (HCFMRP-USP). It consists of a convenience sample of 55 PT-NB of both sexes, with postconceptional age between 32 and 36 weeks and 6 days, hospitalized in the intermediate neonatal care unit (UCIN) of HCFMRP-USP from February 9 to December 9, 2009. After a statistical consult, it was observed that, to proceed to a sample calculation, a single score of the main variable would be necessary. However, the instrument used to assess the neurobehavior (NAPI) of the infants do not have a total score, so the results were expressed only by seven domains.

The inclusion criteria were: infants aged more than five post-natal days, born AGA or SGA according to the classification by Alexander et al*.* ^13^ and clinically stable. In the case of twins, only one infant was selected randomly. The exclusion criteria were: infants presenting with neurological impairment, defined by cephalic perimeter or by transfontanelar ultrasound (intraventricular hemorrhage with ventricular dilation, intraparenchymal hemorrhage, periventricular leukomalacia, hydrocephalus and/or microcephalus); brachial plexus injury; congenital malformation (except for patent ductus arteriosus); suspicion of any genetic syndrome or visual or hearing change; presence of orthopedic changes; suspicion of viral, bacterial or congenital infections; Apgar score £4 on the 5^th^ minute of life; patients under invasive or noninvasive assisted ventilation; NBs who are sedated or in a coma at the time of the evaluation; infants whose mothers had abused illicit toxic substances during pregnancy, except for tobacco and alcohol.

Gestational age (GA) was estimated by ultrasound in the first trimester or using the New Ballard method, and postconceptional age was the sum between GA and chronological age.^14^ The fetal growth curve by Alexander et al*.* ^13^ was used, with P10 and P90 as cutoff points.

The instrument of measurement adopted (NAPI) was translated to Portuguese, with the authorization of the authors, by Formiga, Gabriel and Linhares, and the version in Portuguese was used in a doctoral thesis and in three master’s dissertations, and two of these studies were published.^15^ ^,^ ^16^ NAPI proposes to evaluate the neurobehavioral maturity of the NB with postconceptional age of 32 to 40 weeks regarding motor development and strength, amplitude of passive movements and capacity of attention for auditory and visual stimuli.^10^

The complete evaluation involves 71 items, including physiological signs of the infant and evaluation of the behavioral status of the child, and is carried out in 14 opportunities. Its developmental validity was investigated in seven domains: scarf sign, motor development and vigor, popliteal angle, alert and orientation, irritability, quality of cry and sleep percentage. For the final score, the scores obtained in the seven domains are transformed in the NAPI score, using NAPI’s conversion table, obtaining a 0 to 100 score. The lower the score, the higher the risk of developing problems in the future, except for the domain sleep percentage, which behaves inversely.^10^

The socioeconomic classification involved the criterion of economic classification in Brazil, from the Brazilian Market Research Association (ABEP).^17^ The perinatal aspects of the children included in the investigation were obtained by revising the medical chart, gathering data referring to Apgar in the first and fifth minutes of life; *Clinical Risk Index for Babies* (CRIB I), scored in the first 12 hours of life;^18^ NMI, considering the entire history of hospitalization;^12^ time of hospitalization, clinical complications and clinical measures during the hospitalization, maternal clinical history, conditions of the pregnancy, delivery and birth. Besides, the Röhrer index was calculated,^19^ using weight and length at birth. Symmetrical growth in NB was considered when the Röhrer index was ≥2.49, and the asymmetrical growth was seen in those whose Röhrer index was <2.49.

The inclusion of NB was performed by a collaborator researcher trained by the main researcher, in order to maintain the blind condition of the latter in relation to the groups. Right after the mothers were invited to participate in the study, they were asked to sign the Informed Consent Form; then, the anamnesis was filled out after an interview with the mother, and completed by the reading of the medical chart. The babies were then submitted to an evaluation using NAPI, which occurred in a reserved room to prevent the presence of external noise. Those who were in an incubator on the date of the evaluation were assessed in the unit; however, outside the incubator, at an examination table. In both situations, the mothers were invited to observe the evaluation.

The evaluations were performed exclusively by the main researcher, which generated the reliability of the data. For the control of the prandial condition of the 55 children assessed, the test was carried out from 45 minutes to 1 hour before the children were fed. The evaluation was considered to be at a silent location at the examination room, regardless of the time, or at 8 p.m. in the unit; the noise location included other periods in the same unit. The examination lasted for about 20 minutes and was registered by a collaborator researcher in a recording, using a hand camera HDD, model DCR-SR 220 (Sony, Tokyo, Japan), for posterior analysis.

The obtained data were transferred to a sheet in the Statistical Package for the Social Science for Windows (SPSS), version 15.0 (Chicago, IL, United States). The categorical variables were expressed in number and percentage, and numerical variables in mean, standard deviation, median and maximum and minimum values. The normality of the data was tested by Shapiro-Wilk. When the normal distribution was identified, the continuous variables were compared by the Student’s t-test for independent samples; when the normal distribution was not observed, the distributions were compared by the Mann-Whitney U test. For comparisons between SGA babies with symmetrical growth (n=5) and asymmetrical growth n=27), between symmetrical SGA babies and AGA babies (n=23), and between asymmetrical SGA babies and AGA babies, because of the disproportional size of the sample between groups, the Mann-Whitney U test was also used. The categorical variables were compared using the chi-square or Fisher’s exact test. Because of the statistically significant difference between the SGA and AGA groups regarding post-conceptional age in the evaluation, it was necessary to conduct an adjusted regression analysis to determine the influence of this co-variable in the NAPI results. Significance values were considered as *p*≤0.05 for all tests, and 95% confidence interval (95%CI).

## RESULTS

In the study period, 232 premature babies were initially admitted. Of these, 1 died, 141 presented some of the exclusion criteria, 20 were discharged before there was any time for evaluation, and 15 were lost due to setbacks during the execution of the study. Fifty five NB (61.1%) met all of the criteria and were studied.

Table 1 shows that the babies were born with average GA of 32.0±2.0 weeks, and those born SGA and AGA had similar distribution in relation to gender and frequency of twins. As to post-conceptional age in the evaluation, there was a 1.4 week difference between the SGA and AGA groups (p<0.001). Apgar’s index and CRIB I and NMI severity scores showed good prognosis for the 55 assessed NBs, considering the means and standard-deviations for Apgar at 5 minutes, of 9.3±1.1, for CRIB I, of 1.5±2.3, and for NMI, of 2.3±1.1. There was no statistically significant difference between the SGA and AGA groups in the four analyzed indexes.

**Table 1:** Characteristics of the infants of the total sample and the different groups, as to the adequacy of weight for age.

Characteristics of the babies	Total sample (n=55)	SGA (n=32)	AGA (n=23)	p-value	Test
Weight at birth (grams)					
Média±DP	1491±393	1379±409	1647±317	0,010	Student’s t test
Median (min-max)	1535 (640-2510)	1433 (640-2280)	1565 (1045-2510)		
Gestational age (weeks)					
Mean±SD	32.0±2.0	32.3±2.4	31.7±1.3	0,080	Mann-Whitney U
Median (min-max)	32 (27-36)	33 (27-36)	32 (29-34)		
Post-conceptional age (weeks)					
Mean±SD	34.8±1.3	35.4±1.2	34±1.1	<0,001	Mann-Whitney U
Median (min-max)	35 (32-37)	35.5 (33-37)	34 (32-36)		
Post-natal age (days)					
Mean±SD	19.5±15.1	21.6±18.3	16.5±8.3	0,970	Mann-Whitney U
Median (min-max)	16 (5-67)	15 (5-67)	16 (6-42)		
Twin pregnancy - f (%)	8 (15)	5 (16)	3 (13)	1,000	Fisher’s Exact test
Sex - f (%*)					
Female	30 (55)	18 (56)	12 (52)	0,790	Fisher’s Exact test
Male	25 (45)	14 (44)	11 (48)		
Apgar at 5 minutes (score)					
Mean±SD	9.3±1.1	9.5±1.1	9.1±1.0	0,070	Mann-Whitney U
Median (min-max)	10 (5-10)	10 (5-10)	9 (7-10)		
CRIB I (score)					
Mean±SD	1.5±2.3	1.8±2.8	1.0±1.3	0,500	Mann-Whitney U
Median (min-max)	1 (0-12)	1 (0-12)	1 (0-5)		
NMI (score)					
Mean±SD	2.3±1.1	2.3±1.0	2.4±1.2	0,540	Mann-Whitney U
Median (min-max)	2 (1-5)	2 (1-5)	3 (1-5)		
Time of hospitalization (days)					
Mean±SD	29.0±17.9	31.8±21.2	25.0±11.1	0,540	Mann-Whitney U
Median (min-max)	25 (5-80)	25 (5-80)	26 (7-51)		

SGA: small for gestational age; AGA: adequate for gestational age; SD: standard deviation; f: frequency; %: prevalence; %^*^: percentage; CRIB I: *Clinical Risk Index for Babies*; NMI: *Neonatal Medical Index*.

There were no differences between the groups of infants born SGA and AGA concerning clinical complications during hospitalization and the clinical measures adopted. Likewise, the groups were not different in terms of maternal complications throughout pregnancy or maternal schooling, and the head of the family. The social level of the families, determined by ABEP, mostly corresponded to the C Class (51%), which shows, in Brazil, that these families do not have satisfactory purchasing power and/or that the schooling level of the head of the family is low, once this criterion of economic classification may range between 0 and 34 points, and this class is between 11 and 16 points.

There was no significant statistical difference between the groups concerning the place of evaluation: silent [SGA=24 (75%); AGA=17 (74%); p=1.00], or with noise [SGA=8 (25%); AGA=6 (26%); p=1.00].

There was no statistically significant difference between the SGA and AGA groups, in the analyzed NAPI domains, except for the motor development and vigor domains, which showed better neurobehavioral performance in SGA babies in comparison to AGA infants (Table 2).

**Table 2:** *Neurobehavioral Assessment of the Preterm Infant* (NAPI) scores of the total sample and of the different group, as to the adequacy of weight for gestational age.

NAPI domains	Total sample (n=55)	SGA (n=32)	AGA (n=23)	p-value	Test
Scarf sign					
Mean±SD	41.2±14.3	42.7±15.3	39.1±12.9	0.36	Mann-Whitney U
Median (min-max)	33.3 (33.3-66.7)	33.3 (33.3-66.7)	33.3 (33.3-66.7)		
Motor and vigor					
Mean±SD	49.5±14.3	52.7±14.6	45.1±13.0	0.05	Student’s t test
Median (min-max)	49.2 (19.6-87.5)	50.9 (24.7-87.5)	42.6 (19.6-68.1)		
Popliteal angle					
Mean±SD	60.5±30.4	63.5±33.2	56.1±26.0^a^	0.29	Mann-Whitney U
Median (min-max)	66.7 (0.0-100.0)	66.7 (0.0-100.0)	50.0 (33.3-100.0)		
Alert and orientation					
Mean±SD	62.7±13.9	62.9±13.9	62.5±14.2	0.94	Mann-Whitney U
Median (min-max)	67.0 (29.3-80.4)	66.6 (29.3-80.4)	67.8 (31.9-79.3)		
Irritability					
Mean±SD	42.3±19.6	42.7±19.2	41.8±20.5	0.84	Mann-Whitney U
Median (min-max)	36.9 (0.0-71.5)	43.5 (0.0-64.3)	36.9 (0.0-71.5)		
Cry					
Mean±SD	39.4±34.5	37.5±35.0^b^	42.1±34.4^c^	0.64	Mann-Whitney U
Median (min-max)	50.0 (0.0-100.0)	50.0 (0.0-100.0)	50.0 (0.0-100.0)		
Sleep percentage					
Mean±SD	48.2±24.0	47.3±27.0	49.4±19.5	0.76	Student’s t test
Median (min-max)	50.0 (0.0-100.0)	50.0 (0.0-100.0)	50.0 (0.0-100.0)		

NAPI: *Neurobehavioral Assessment of the Preterm Infant*; SGA: small for gestational age; AGA: adequate for gestational age; SD: standard deviation;.^a^: n 22; ^b^: n 28; ^c^: n 19.

Post-conceptional age was significantly different between groups: babies born SGA had more advanced age in comparison to babies AGA. So, it was necessary to carry out a secondary analysis to determine the influence of this co-variable over the domain motor development and vigor. This post-analysis (adjusted regression analysis) showed no significant difference between the groups in this NAPI domain, which in fact suggests that post-conceptional age was influencing the data as a confounding factor. Therefore, the group of infants born SGA presented similar performance to the group of infants born AGA, also in the domain motor development and vigor, as shown in Table 3.

**Table 3:** Influence of post-conceptional age in the motor development and vigor domain.

	Estimation of the effect	95%CI	p-value
SGA × AGA groups	-6.33	-15.36-2.69	0.17
Post-conceptional age	0.95	-2.48-4.38	0.58

SGA: small for gestational age; AGA: adequate for gestational age.

Regarding the comparisons between babies SGA of symmetrical growth and babies with asymmetrical growth, between symmetrical SGA and AGA, and between the asymmetrical SGA and AGA, once again, there was no difference in none of the NAPI domains. However, when some of the co-variables were tested considering these subgroups, some statistically significant differences were observed, as seen in Table 4.

**Table 4:** Characteristics of the infants according to the adequacy of weight for gestational age and type of growth.

Characteristics of the babies	Symmetrical SGA (n=5)	Asymmetrical SGA (n=27)	AGA (n=23)	p-value (sim SGA × asym)	p-value (sym SGA × AGA)	p-value (asym SGA × AGA)
Weight at birth (grams)						
Mean±SD	1665±252	1326±413	1647±317	0.08	0.88	0.01
Median (min-max)	1630 (1430-2065)	1350 (640-2280)	1565 (1045-2510)			
Gestational age (weeks)						
Mean±SD	33.8±1.3	32.0±2.5	31.7±1.3	0.10	0.01	0.26
Median (min-max)	34 (32-35)	33 (27-36)	32 (29-34)			
Post-conceptional age (weeks)						
Mean±SD	35.4±1.5	35.4±1.1	34.0±1.1	0.79	0.04	0.00
Median (min-max)	36 (33-37)	35 (33-37)	34 (32-36)			
Post-natal age (days)						
Mean±SD	9.8±3.8	23.8±19.1	16.5±8.3	0.05	0.06	0.57
Median (min-max)	9 (6-16)	16 (5-67)	16 (6-42)			
CRIB I (score)						
Mean±SD	0.4±0.5	2.0±2.9	1.0±1.3	0.17	0.35	0.29
Median (min-max)	0 (0-1)	1 (0-12)	1 (0-5)			
NMI (score)						
Mean±SD	1.4±0.5	2.4±1.0	2.4±1.2	0.03	0.05	0.96
Median (min-max)	1 (1-2)	2 (1-5)	3 (1-5)			
Number of respiratory complications						
Mean±SD	0.6±0.5	1.7±1.7	1.5±0.9	0.21	0.03	0.64
Median (min-max)	1 (0-1)	1 (0-7)	2 (0-3)			
Time of hospitalization in NICU (days)						
Mean±SD	1.4±2.2	12.9±18.2	6.7±6.7	0.05	0.06	0.67
Median (min-max)	0 (0-5)	6 (0-60)	4 (0-27)			

SGA: small for gestational age; AGA: adequate for gestational age; SD: standard deviation; sym: symmetrical; asym: asymmetrical; CRIB I: *Clinical Risk Index for Babies*; NMI: *Neonatal Medical Index*; NICU: neonatal intensive care unit.

However, when some of the co-variables were tested considering these subgroups, there were some statistically significant differences, as observed in Table 4. The infants SGA of symmetrical growth, when compared to babies SGA of asymmetrical growth, presented lower score in NMI (lower clinical risk), lower time of hospitalization in the neonatal ICU, besides having been assessed by NAPI at a younger post-natal age.

In comparison to babies born AGA, the group of SGA of symmetrical growth had higher GA, lower score in the NMI, fewer respiratory complications, and was assessed with NAPI at a more advanced post-conceptional age. However, the evaluation was carried out at an inferior post-natal age, with p value close to that of the statistical significance. Finally, when the group of babies SMA of asymmetrical growth was compared to that of babies AGA, there was lower weight at birth and higher post-conceptional age in the NAPI evaluation.

## DISCUSSION

There was no statistically significant difference in the NAPI domains between the groups of infants born SGA and AGA.

We do not know of any study comparing the neurodevelopment before term in premature infants born SGA and AGA, like this study. Feldman and Eidelman^7^ studied 120 premature NBs from single pregnancies, being 40 SGA in group 1 compared tot wo other control groups, which were: group 2, composed of 40 AGA paired by weight at birth; and group 3, composed of 40 AGA paired by AG. For the analysis, the three groups were divided by weight at birth below or above 1,000 g. These authors verified that newborns SGA presented with unfavorable neuropsychomotor development throughout childhood, including poor organization skills and neurobehavioral maturation, especially in the motor and orientation domains, assessed by the *Neonatal Behavioral Assessment Scale* (NBAS).^20^ These children also presented with impaired social behavior throughout childhood, as well as impaired cognitive development at the age of one and two years old, assessed with BSID II.^21^ The NB SGA with weight at birth <1,000 g had significantly lower scores in relation to the other groups. The authors then concluded that NBs SGA have double risk (condition of being SGA and weight at birth <1.000 g) for delay in neuropsychomotor development; however, the mentioned study assessed development at age of term, and during the first childhood, which is different from the evaluation in this study.

There is speculation that the absence of difference in neurobehavior, assessed by the NAPI, among preterm infants SGA and AGA was owed to the fact that they were all in accordance with a narrow range of variation of gestational and post-conceptional age, insufficient to detect significant differences in neuropsychomotor development.

In this study, even though infants SGA with symmetrical growth were younger in relation to post-natal age, compared to infants SGA of asymmetrical growth and AGA, the number of individuals in this subgroup of patients was reduced, in comparison to other subgroups. Therefore, it was not possible to formulate any hypothesis regarding the neurobehavioral findings.

So, it is possible to infer that age in the evaluation with the NAPI had a direct influence on the neurobehavioral maturity of infants in the study, and that, probably, the lack of differences in the neurobehavior among babies born premature SGA and AGA is not a result of the condition of adequate weight for gestational age. In this sense, it is important to mention that babies with major neurological changes were excluded from this study in order to understand the influence of being premature SGA or AGA in neurobehavior. It is important to mention there was no previous randomization of the groups and that the division took place a posteriori, in order to verify for possible neurobehavioral differences related to the adaptation for gestational age at first.

Despite the predictive validity of the NAPI, which was observed in other studies, it still has not been tested in studies comparing groups of premature babies born SGA and AGA at the initial stage of neurodevelopment. Since this was not the focus of this study, further studies with a longitudinal design are suggested in order to verify if these groups would continue to present similar neurodevelopment.

The limitations of this study were the type of convenience sample, with small sample size, and the cross-sectional design, which did not allow the long-term knowledge of the neurodevelopment of the babies. Besides, the findings cannot be generalized to any sample of premature infants, because of the inclusion and exclusion criteria of this study.
